# Infantile Anorexia and Co-parenting: A Pilot Study on Mother–Father–Child Triadic Interactions during Feeding and Play

**DOI:** 10.3389/fpsyg.2017.00376

**Published:** 2017-03-17

**Authors:** Loredana Lucarelli, Massimo Ammaniti, Alessio Porreca, Alessandra Simonelli

**Affiliations:** ^1^Department of Pedagogy, Psychology, Philosophy, University of CagliariCagliari, Italy; ^2^Faculty of Medicine and Psychology, Sapienza University of RomeRome, Italy; ^3^Department of Developmental and Social Psychology, University of PadovaPadova, Italy

**Keywords:** infantile anorexia, co-parental and family subsystems, mother-father-infant feeding and play interactions, clinical assessment and treatment

## Abstract

Infantile Anorexia (IA), defined by the Diagnostic Classification of Mental Health and Developmental Disorders of Infancy and Early Childhood Revised (DC: 0-3R, Zero To Three, 2005), occurs when the child (a) refuses to eat adequate amounts of food for at least 1 month, and shows growth deficiency, (b) does not communicate hunger and lacks interest in food, and (c) the child’s food refusal does not follow a traumatic event and is not due to an underlying medical illness. IA usually emerges during the transition to self-feeding, when the child issues of autonomy are played out daily in the feeding situation. Studies evidence that the feeding interactions between children with IA and their mothers are characterized by low reciprocity, greater interactional conflict and negative affects (Chatoor et al., 2000; Ammaniti et al., 2010, 2012). Moreover, these studies pointed out that maternal depression and eating disorders are frequently associated with IA (Cooper et al., 2004; Ammaniti et al., 2010; Lucarelli et al., 2013). To date, research has focused almost exclusively on the mother–child dyad, while fathers’ involvement, co-parental and family interactions are poorly studied. The current study is a pilot research that investigated mother–father–child triadic interactions, during feeding and play, in families with children diagnosed with IA, in comparison to families with normally developing children. Until now, at the study participated *N* = 10 families (five with a child with IA diagnosis and five with lack of child’s IA diagnosis, matched for child’s age and gender). The parents–child triadic interactions were assessed in feeding and play contexts using the Lausanne Trilogue Play (Fivaz-Depeursinge and Corboz-Warnery, 1999), adapted to observe father-mother-infant primary triangle in the feeding context, compared to the play context (Lucarelli et al., 2012). Families of the IA-group showed difficulties in expressing and sharing pleasure and positive affects, and in structuring a predictable and flexible context. Children showed little autonomy and difficulty in being actively engaged and tune with parents. Dysfunctional family interactions are a critical issue for IA that affects co-parental and family subsystems, stressing the importance of an articulated diagnostic assessment in order to target effective treatment approaches.

## Introduction

### Assessing Adult–Child Interactions: A Shift toward a Triadic Perspective

In the recent years, there has been an increasing interest in the clinical and scientific investigation of interactive contexts which are more complex than the dyadic one. This progressively led to focus the attention on the family triadic system, conceptualized as a primary interactive and relational matrix, parallel and not subsequent to the dyadic system, where the child can develop his/her socio-emotional abilities (Simonelli et al., 2014). It has been pointed out that triadic interactive competencies seem to emerge very early during infancy, following a developmental trajectory that is parallel to the one related to the development of dyadic interactive competencies. Several studies suggested the presence of a form of early intersubjectivity in young children which is not exclusively ascribable to the intersubjectivity emerging from dyadic adult-child interactions (Nadel and Tremblay-Leveau, 1999; Fivaz-Depeursinge et al., 2005). For example, at 3–4 months, babies already show the ability to coordinate their attention and affects between two partners simultaneously, tracking back-and-forth exchanges between two adult partners and making triangular bids that allow them to share attention and affects (Fivaz-Depeursinge and Corboz-Warnery, 1999). These precocious “triangular capacities” are not only scaffolded by the adults, but also seem to emerge from the babies’ own initiatives, even in the absence of the adult bids (McHale et al., 2008). During the interactive exchanges with his/her care-givers, the child gradually becomes more skilled in experiencing “schemas-of-being-with” another person (Stern, 1985) that allow to organize the interactive history into triadic schemas which involve the repeated experience of being simultaneously in interaction with more than one person per time (Fivaz-Depeursinge and Corboz-Warnery, 1999). Thus, according to this perspective, the quality of the triadic interactions and the development of triadic competencies are no longer considered as a developmental step subsequent to the acquisition of dyadic interactive competencies. Rather, they appear since early infancy and become already observable during the first months of life (Tremblay-Leveau and Nadel, 1995; Fivaz-Depeursinge and Corboz-Warnery, 1999; Tremblay-Leveau, 1999).

Moreover, different studies pointed out that these early triangular capacities are more likely to develop functionally in the context of family alliance, which is defined as the quality of the interactive coordination between family members (McHale et al., 2008; Favez et al., 2012). In this sense, the quality of family alliance constitutes the context for the child to learn emotion regulation and to develop an understanding of inner states (Favez et al., 2012). Another aspect linked with the adoption of a triadic perspective in the investigation of a family functioning and of a child development concerns co-parenting, conceptualized as the extent to which partners share leadership and support each other in their roles as architects and heads of the family (Minuchin, 1974; McHale, 1995). Although parents may provide very different interpersonal experiences for the baby, when co-parenting is functional the partners cooperate and coordinate together, supporting each other’s efforts, and accommodating their individual styles and preferences during care-taking practices (McHale et al., 2008). Otherwise, opposition and detachment might arise, with one parent interfering with the partner’s attempts, or through the collusive transference of most effort to one parent to the exclusion of the other (McHale et al., 2008). It has been shown that the early interactions assessed at a family level and co-parenting patterns present consistent associations with the child’s socio-emotional development, showing that different family alliances constitute different contexts, which are more or less optimal for the infant development of emotion regulation (McHale, 2007; Favez et al., 2012; Simonelli et al., 2012).

Together with the infant’s early triangular capacities, co-parenting contributes in determining the quality of the family functioning that, although to some extent maintained through mental representations, is primarily “practiced” through everyday interactive exchanges between infant and parents (Fivaz-Depeursinge and Corboz-Warnery, 1999). From this perspective, the investigation of observable behaviors constitutes a preferential way to achieve a better understanding of the family system and of the early adult–child relationships (Fivaz-Depeursinge et al., 2010; Simonelli et al., 2010, 2012, 2014). Moreover, this kind of investigation could help to deepen the knowledge of family functioning further beyond early infancy, as suggested by a recent longitudinal study on the Italian population, carried out from pregnancy to the preschool age period, which highlighted the presence of developmental trends in the quality of family interactions (Simonelli et al., 2016).

### The Role of Adult–Child Interactions in Infantile Anorexia

While early feeding disturbances are quite frequent in the infantile population, affecting around 25–35% of children (Lyons-Ruth et al., 1996; Benoit, 2000; Chatoor, 2009), feeding disorders are less frequent and specifically characterized by inadequate intake of food expresses as *failure to thrive* or *growth stunting;* the child does not regulate feeding in accordance with physiological feelings of hunger or fullness, showing difficulties in establishing regular feeding patterns. Regarding the pathogenesis of early feeding disorders, it is estimated that malnutrition and failure to thrive may include both organic and non-organic factors, but only 16–30% have their origin in an organic disease that might explain growth problems (Benoit, 2000). Some authors found associations between feeding and eating problems in infancy and disturbances in the mother–infant relationship, suggesting to consider feeding disorders as relationship or transactional disorders (Goodlin-Jones and Anders, 2001; Chatoor et al., 2004; Feldman, 2007). In line with these results, clinical studies and research have pointed out that diagnostic criteria for early feeding disorders which focus only on the infant fail to incorporate the relationship phenomenon linking dynamics of the caregiving environment with the infant feeding disorder (Chatoor, 2009; Ammaniti et al., 2010; Bryant-Waugh et al., 2010). In fact, in early childhood, feeding represents a pivotal experience for the development of the relationship between mother and child, in which emotional signals and the sharing of affects promote the communication of needs, desires and pleasure, as well the stabilization of biological rhythms (Stern, 1996, 2010; Lieberman and Slade, 2000; Feldman, 2007; Fadda et al., 2014; Murray, 2014). Feeding thus develops a close emotional engagement and a “conversational” setting, where the parents make sense of their baby’s expressiveness and communicate their empathy and understanding, laying the foundations for affective and social communication. As he/she grows up, the infant needs to reach more physical and emotional independence, through a process that requires to reach an equilibrium between attachment to the caregiver and emerging autonomy, according to age and developmental stage. Parallel to this developmental task, the parents are engaged in a process that involves the progressive balancing of protective behaviors and “letting go” behaviors, which stimulate feeding self-regulatory abilities, autonomous initiatives and the self-reliance of the child.

Therefore, the relationship between the mother and the child is characterized by a high degree of coordination and the exchanges constitute a system of interactive regulation, in which each partner influences and regulates the behavior of the other. These influences include favoring or blocking reciprocal adaptation, protecting from possible risk factors or, on the contrary, transmitting negative influences. Clinical distortions in the relationship may occur when mother and child are locked into a rigid stance where empathic communication breaks down and neither partner can understand or cooperate with the emotional or developmental agenda of the other (Fonagy et al., 2002/2005; Feldman, 2007; Ammaniti and Gallese, 2014).

This study aims to explore the feeding disorder subtype of IA, defined by the *Diagnostic Classification of Mental Health and Developmental Disorders of Infancy and Early Childhood Revised* (DC: 0-3R, Zero To Three, 2005). IA usually becomes evident before the child is 3 years old, when young children are transitioned to self-feeding, and when issues of autonomy and dependency have to be negotiated between parents and child. Toddlers with IA have been found to have a higher level of physiological arousal and more difficulty down-regulating their arousal (Chatoor et al., 2004). However, studies have found significant correlations among difficult child temperament, irregular feeding and sleeping patterns, negative and willful behaviors by the toddlers, and mother–child interactional conflict, low dyadic reciprocity and negative affects during feeding (Stein et al., 1999; Chatoor et al., 2000; Ammaniti et al., 2004a, 2010; Lucarelli et al., 2013). At the same time, mothers’ insecure attachment to their own parents and mothers’ drive for thinness and bulimia, anxiety, and depression also correlated significantly with mother–child conflict during feeding (Stein et al., 1999; Chatoor et al., 2000; Ammaniti et al., 2004b, 2010; Cooper et al., 2004; Lucarelli and Speranza, 2014). These results were confirmed also in the case of maternal binge eating disorders, where the presence of dysfunctions in mother–child feeding interactions was found to significantly influence and mediate the impact of maternal psychopathology on later child emotional and behavioral difficulties (Cimino et al., 2016). Studies also showed that the mother–child conflict during feeding correlated strongly with the child’s weight, indicating that a higher conflict between mother and child was associated with lower child’s weight (Chatoor et al., 2000). More recently, research evidenced that the quality of maternal models of attachment is a significant predictor of the severity of malnutrition in the children diagnosed with IA (Lucarelli and Speranza, 2014), further confirming the role of mother-child interactions in the etiology and evolution of feeding disturbances and disorders during infancy.

Overall, these studies confirm that to date research about IA has focused almost exclusively on the mother–child dyad, while fathers’ involvement, co-parental and family interactions are poorly studied. Yet, studies on non-referred samples have shown that the level of father involvement in childcare predicts the quality of family interactions from the earliest stages of the child’s life (Simonelli et al., 2016). Moreover, clinical data and some early research on infant feeding disorders suggest to consider the impact of the father’s role in mother–child caregiving and co-parenting systems, highlighting more difficulty of the fathers in supporting the mother–child caregiving system in the process of the child’s regulation of eating and affective differentiation (Barriguete et al., 2002). Moreover, less than optimal quality of maternal and paternal dyadic interactions during feeding and play, higher maternal involvement and lower paternal involvement in child care, compared with controls, have been found in families with a child diagnosed with a non-organic-based food refusal (Atzaba-Poria et al., 2010).

The current study is a new pilot research which aims to investigate mother–father–child triadic interactions during feeding and play in families with children diagnosed with IA. In order to achieve this purpose, the procedure of the Lausanne Trilogue Play (LTP, Fivaz-Depeursinge and Corboz-Warnery, 1999) was applied to these families both in its original format, developed by the authors to investigate the quality of mother–father–child free play interactions, and in a format adapted to the feeding situation, in order to assess triadic mother–father–child feeding exchanges. The adaptation of the LTP procedure to the feeding situation was guided by the objective to observe and to assess the presence of differences in family interactive style between the play and the feeding condition, in families with children with IA and in families with children showing typical development. In both cases (play and feeding interactions) the theoretical and empirical framework is based on recent findings about the co-parenting and early intersubjectivity that have highlighted how the infants use their social competence very early to communicate not only in dyads but also in triads, particularly in the triangle they form with their mother and father (Fivaz-Depeursinge and Corboz-Warnery, 1999; Carneiro et al., 2006). Family alliance is defined as the quality of the interactive coordination between family members and constitutes a context for the child to learn emotion regulation and to develop an understanding of inner states. In this perspective, the development of this triangular communication is largely shaped by the ways the parents support or undermine each other in relation to their child (Fivaz-Depeursinge, 2008; Favez et al., 2012).

## Aims and Hypotheses

The main objective of this pilot study was to investigate the quality of mother–father–child triadic interactions in the contexts of Feeding and Play in families whose children were diagnosed with IA, and to compare them with the results of families with normally developing children. For these purposes we adopted the LTP (Fivaz-Depeursinge and Corboz-Warnery, 1999), a procedure developed to assess triadic interactions. The procedure was administered both in its original version, for the assessment of triadic interactions during play, and in a version where setting and assignments were modified in order to evaluate triadic exchanges in the context of feeding. In this sense, a complementary aim of our pilot study was to verify the applicability of the LTP to the Feeding context; more specifically we aimed to verify the feasibility to apply the LTP settings and assignments (i.e., the task to interact in a triadic way assuming a specific spatial and bodily arrangement) to the feeding context and to see whether this adaptation was tolerated by the families. With respect to the clinical hypotheses of our work, according to the literature on IA, we expected to find lower quality triadic interactions during Feeding (a) and during Play (b) in the IA-Group with respect to the control group, as suggested by studies highlighting higher difficulties in mother–child and father–child interactions in families with a child feeding disorder (Atzaba-Poria et al., 2010). Moreover, far as it concerns specifically the IA-Group, we expected to find more difficulties in triadic interactions during the Feeding context rather than the Play context (c); this hypothesis was guided by the assumption that interactive difficulties in families with children diagnosed with IA would be more likely to involve the specific domain of feeding rather than other interactive domains.

## Materials and Methods

### Participants

The study involved five families with children diagnosed with Infantile Anorexia (IA-Group), extracted from a larger group of 51 families with a child diagnosed with IA (Lucarelli et al., 2013) in a Pediatric Hospital of Rome (Italy). The IA-Group was selected through a clinical and diagnostic assessment which excluded the presence of current organic causes as the origin of children’s difficulties in establishing regular feeding rhythms and an intake of adequate amounts of food, evaluating the presence of child’s malnutrition, based on the weight and height measurements from the National Center for Health Statistics’ Growth Charts using Waterlow’s criteria (Waterlow et al., 1977; Kuczmarski et al., 2000). The diagnosis was made independently by two clinicians (*k* = 0.93), on basis of the criteria of the DC:03-R (Zero To Three, 2005).

In the subsample of five families investigated in this study, the children’s mean age was 42.6 months (*SD* = 2.97) while the parents’ mean ages were respectively 35 (*SD* = 4) and 39 (*SD* = 4.5) years for the mothers and for the fathers. Families were assessed during two observational procedures aimed at investigating the quality of mother–father–child triadic interactions. The results were then compared with a control group (CTRL Group), composed by non-referred parents and normally developing children, extracted from a larger research project (Simonelli et al., 2014, 2016). The research described here was approved by the Academic Institutional Board, and in accordance with the Declaration of Helsinki. Written informed consent was obtained from each of the study participants.

### Procedures

All the families were observed and video-recorded during the Lausanne Trilogue Play (LTP, Fivaz-Depeursinge and Corboz-Warnery, 1999), a semi-standardized procedure developed to assess the quality of mother–father–child triadic interactions. The procedure follows a four-part scenario: during the first part (2 + 1) one of the parents interacts with the child, while the other one remains in the position of third party; during the second part (2 + 1) the parents reverse their roles; during the third part (3-together) all the family members interact together; finally, during the fourth part (Parents’ dialog) the parents are asked to talk together while the child remains in the position of third party. In this way, the procedure covers all the possible configurations that a three-person interaction could assume. The parents are informed that the procedure usually takes about 10–15 min, but are left free to decide the effective duration of the scenario, the duration of each part, and who should begin to interact with the child. For the purposes of this study, the LTP procedure was administered both in its original version, developed to assess triadic interactions during Play, and in a version adapted to the Feeding context, where the four-part structure remained unvaried but the parents were asked to bring a meal for the child. Parents were asked to bring the type of food they usually offered their children and behave as they usually did with their children at home following the four-part scenario described above, i.e., during the first part (2 + 1) one of the parents had to carry out the meal with the child, with the other parent remaining third party; during the second part (2 + 1) the parents switched their roles; during the third part (3-together) all the family members carried out the meal together; finally, during the fourth part (Parents’ dialog) the parents were asked to talk together while the child was supposed to eat by himself/herself. Again, the parents were informed that the procedure lasted about 10–15 min and where left free to decide who should begin, the duration of each part, and the effective duration of the procedure. The adaptations of the setting and of the assignments of the LTP to the feeding situation were discussed with the authors until the realization of the actual arrangement, which was approved for the investigation of these families with a child with IA.

### Measure

#### Family Alliance Assessment Scales

The families were evaluated during the LTP with the Family Alliance Assessment Scales (FAAS; Favez et al., 2011; Lavanchy Scaiola et al., 2008, unpublished), an observational tool for the assessment of father–mother–child interactions that considers different scales related to specific dynamics of family interactions which could be scored as “appropriate,” “moderate” and “inappropriate.” More specifically, the scales refer to^1^:

(1) Postures and gazes which are optimal to create a context that enhances emotional exchanges and the sharing of affects in the family.
(2) Inclusion of the partners in the play, and ability to take each other into account.
(3) Implication of each partner in his role, engaging in interactions when they are supposed to be active and non-interfering in the other partner’s activities when they are supposed to be the third party.
(4) Respect for the task’s structure and timeframe, carrying out and maintaining distinct the four parts of the scenario, with each part lasing enough for a joint activity to be set up but at the same time adjusted to the child’s state and aligned with the progression of the procedure.
(5) Co-construction of a joint activity.
(6) Parental scaffolding, with stimulations adapted to the child’s age and state.
(7) Family warmth, with positive affects circulating through and shared by all partners and with an empathetic attitude shown with respect to negative affects.
(8) Validation of the child’s emotional experience and sensitivity to the child’s cues.
(9) Authenticity of the expressed affects, which are supposed to be congruent with the situation and coherent with respect to the behaviors and the affects expressed by the other partners.
(10) Interactive mistakes and their resolution during activities, so that the interaction could maintain its flow and that the partners are able to smoothly carry out effective resolutions to the possible interactive mistakes.
(11) Interactive mistakes and their resolution during transitions, carrying out the transition from one configuration to another in a fluid way, announcing the change, implicitly or explicitly, and negotiating the transition.
(12) Support and cooperation between the parents, with mutual verbal and non-verbal support.
(13) Conflicts and disruptive interferences in co-parental coordination
(14) Child’s involvement and ability to be engaged in the interaction.
(15) Child’s self-regulation

All the videos were coded by two independent judges trained and reliable to the use of the FAAS, who were blind to the children’s IA diagnosis. The application of the LTP reported good inter-rater reliability, ranging from *r* = 0.81 to *r* = 0.97. The application of Cronbach’s alpha coefficient reported acceptable internal consistency in all the variables considered by the coding system of the LTP procedure, both in the Play (0.737 < α < 0.761) and in the Feeding interactional contexts (0.753 < α < 0.767). More specifically, as far as it concerns the application of the LTP to the feeding situation, the presence of such good indexes of internal consistency in all the variables considered appears to support the suitability of the application of the procedure to contexts which are different from the original one represented by the play situation. This result appears in line with previous studies that adapted the LTP procedure according to family culture, children’s age, peculiarities in development, and which did not result to compromise the internal consistency and the reliability of the coding system (Hedenbro et al., 2006; Miscioscia et al., 2013; Gatta et al., 2014, 2015).

## Results

### Comparison between the IA-Group and the Control Group

#### Triadic Interactions in Feeding Context

**FIGURE 1:**
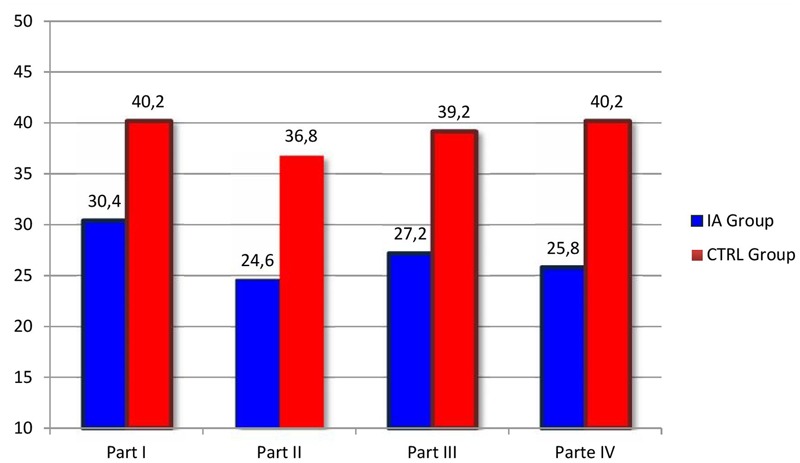
**Comparison between the four parts of the LTP in the IA Group and in the CTRL Group during the Feeding context.** The columns in evidence indicate the LTP parts where statistically significant differences between the two groups were found.

**FIGURE 2:**
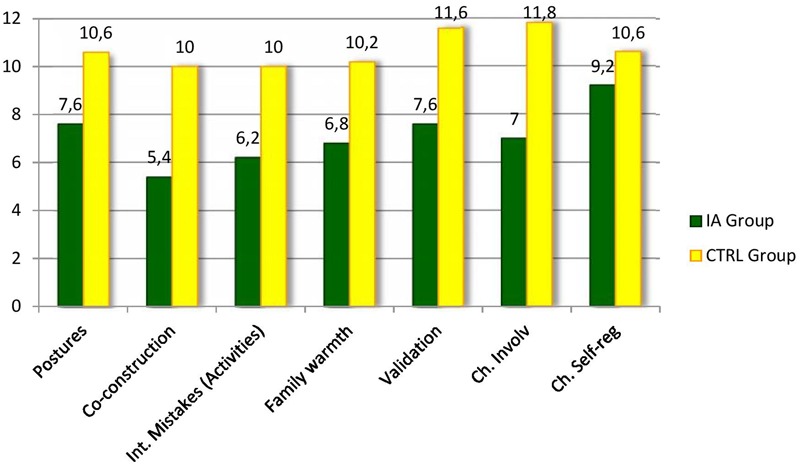
**Family Alliance Assessment Scales (FAAS) where the IA Group and the CTRL Group showed significant differences during the Feeding context**.

The Mann–Whitney *U*-test for independent samples was applied to compare the scores of the IA-Group and of CTRL-Group at the LTP procedure in the Feeding context. Analyses were run both on the global scores of each part and on the scores of each of the interactive variables considered by the coding procedure. **Figures 1, 2** report the results concerning respectively the comparison between the four parts of the LTP and the FAAS variables where the IA Group and the CTRL Group showed significant differences in family interactions in the context of feeding. As it is possible to see, as far as it concerns the comparison of the four parts, the results showed statistically significant differences in three of the four parts constituting the procedure. More specifically, significant differences emerged with respect to the first part (2 + 1) (*Z* = -2.312, *p* < 0.05), the third part (3-together) (*Z* = -2.694, *p* < 0.05), and the fourth part (Parents’ dialog) (*Z* = -2.635, *p* < 0.05) (**Figure 1**). As far as it concerns the specific interactive dimensions, statistically significant differences were found concerning several FAAS variables. Specifically, the differences between the two groups emerged in Postures and gazes (*Z* = -2.69, *p* < 0.05), Co-construction (*Z* = -2.65, *p* < 0.05), Interactive mistakes and their resolution during activities (*Z* = -2.54, *p* < 0.05), Family warmth (*Z* = -2.69, *p* < 0.05), Validation of the child’s emotional experience (*Z* = -2.65, *p* < 0.05), Child’s involvement (*Z* = -2.71, *p* < 0.05) and Child’s self-regulation (*Z* = 2.45, *p* < 0.05) (**Figure 2**). Thus, it seemed that during the Feeding situation the parents of the IA-Group showed more difficulties in adopting gazes and a body orientation adequate to create an optimal context for the interaction, in co-constructing a joint activity and in carrying out adequate resolutions of the possible interactive mistakes emerging during shared activities. Moreover, the IA families seemed to experience more difficulties in sharing circularly positive emotions and in being sensitive and validating toward the children’s affective experience. On the other hand, children diagnosed with IA resulted less engaged during interactions with their parents and showed less competencies in self-regulation.

#### Triadic Interactions in Play Context

**FIGURE 3:**
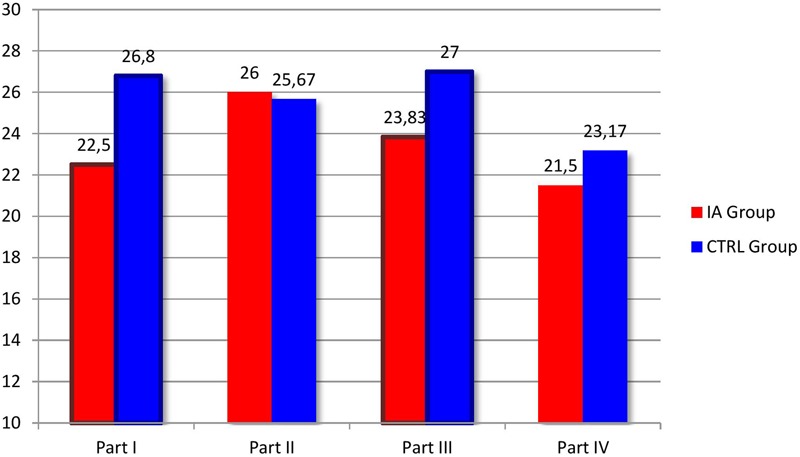
**Comparison between the four parts of the LTP in the IA Group and in the CTRL Group during the Play context.** The columns in evidence indicate the LTP parts where statistically significant differences between the two groups were found.

**FIGURE 4:**
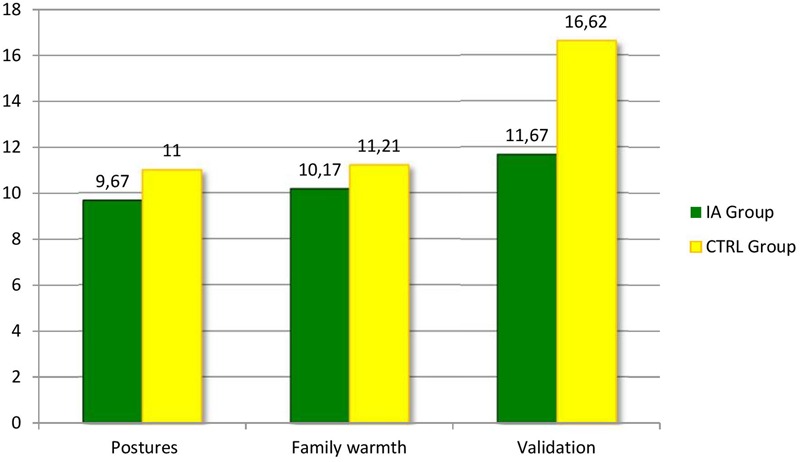
**Family Alliance Assessment Scales where the IA Group and the CTRL Group showed significant differences during the Play context**.

The Mann–Whitney *U*-test for independent samples was applied to compare the scores of the IA-Group and the CTRL-Group at the LTP procedure in the Play context. The analyses were run both on the global scores of each part and on the scores of each of the interactive variables considered by the coding procedure. **Figures 3, 4** report the results of the comparisons between the four parts of the LTP and the FAAS variables where the IA Group and the CTRL Group showed significant differences in the context of Play. As it is possible to see, the Play context reported less differences than the Feeding one. More specifically, as far as it concerns the comparison of the four parts, the results showed statistically significant differences concerning the first (2 + 1) (*Z* = -3.53, *p* < 0.05) and the third part (3- together) (*Z* = -2.40, *p* < 0.05) (**Figure 3**). As far as it concerns the more specific interactive variables, differences were found concerning Postures and gazes (*Z* = -2.43, *p* < 0.05), Family warmth (*Z* = -2.51, *p* < 0.05) and Validation of the child’s emotional experience (*Z* = -2.79, *p* < 0.05) (**Figure 4**). Also during Play families whose children were diagnosed with IA seemed to experience more difficulties in adopting gazes and a body orientation adequate to create an optimal context for the interaction, in sharing positive emotions and in being sensitive and validating toward the children’s affective experience.

### Comparison between Feeding and Play Triadic Interactions in the IA-Group

The Wilcoxon Signed-Rank test was applied to investigate whether the IA-families experienced more difficulties in triadic interactions during Feeding or during Play. The results showed statistically significant differences regarding several LTP variables, as far as it concerns Postures and gazes (*Z* = -2.03, *p* < 0.05), Implication of each partner in his role (*Z* = -2.03, *p* < 0.05), Co-construction (*Z* = -2.03, *p* < 0.05), Family Warmth (*Z* = -2.04, *p* < 0.05), Validation of the child’s emotional experience (*Z* = -2.03, *p* < 0.05), Support (*Z* = -2.03, *p* < 0.05) and Child’s involvement (*Z* = -2.07, *p* < 0.05), with lower scores in the Feeding situation.

## Discussion

The basic issue of our study was to empirically support a transactional and multi-risk model for IA (Chatoor, 2009; Ammaniti et al., 2010, 2012; Sameroff, 2010; Lucarelli et al., 2012, 2013). Recent literature highlighted the presence of associations between fathers’ involvement and feeding disorders, indicating the need to move beyond previous theories which focused exclusively on the mother-child dyad to explain the aetiogenesis and the development of child psychopathology (Barriguete et al., 2002; Atzaba-Poria et al., 2010; Hall et al., 2014). According to this literature, we suggest a model where difficulties in feeding, and more generally in adult–child interactions, might influence and be influenced in a specific way by the functioning of the broader triadic mother–father–child system, beyond the one of the single mother–child and father–child dyadic subsystems.

From this perspective, we investigated triadic functioning in families whose children were diagnosed with IA, comparing the results with the ones of families with normally developing children. This was done both in the context of Feeding and Play through the application of the LTP (Fivaz-Depeursinge and Corboz-Warnery, 1999), a procedure specifically developed to assess the quality of mother–father–child triadic interactions, coded through the FAAS (Lavanchy Scaiola et al., 2008, unpublished), which takes into account different levels of family interactive functioning.

Our two first hypotheses were to find in the IA-Group triadic interactions of lower quality during (a) the condition of Feeding and (b) the condition of Play, with respect to the CTRL-Group. These hypotheses were both confirmed, with significant differences on the LTP global scores of the parts and on the single FAAS variables in both situations. Moreover, given the specific clinical dysfunction of feeding patterns in IA, we made a third hypothesis (c) with specific reference to the IA-Group, expecting to find significantly lower scores in triadic interactions in the Feeding condition rather than in the Play condition. As expected, the results confirmed this hypothesis, with significant differences on several FAAS variables. Although keeping in mind the limited possibility of generalization due to the small sample of families, some considerations could be made about these results.

First, what it is interesting to observe is that the IA-Group generally showed triadic mother–father–child interactions of lower quality with respect to the CTRL Group. Anyway, the Feeding context showed a higher number of significant differences, suggesting the presence of specific interactive difficulties in families with children diagnosed with IA.

Secondly, another remarkable result is that both Feeding and Play interactional contexts highlighted significant differences in the LTP global scores of the first and the third part. In the LTP, the first part is the one where the family needs to negotiate and to organize the beginning of the procedure. In optimal situations, the activity is expected to be announced and to begin with a smooth organization of the interaction, characterized by quick and resolved negotiations, both explicitly and implicitly. This step seemed particularly difficult for the IA-Group. In family 1 and family 4, for example, it was possible to observe that one parent took the initiative in a unilateral way, without announcing it and without negotiating it with the partner, whereas family 2 seemed to show a general clumsiness, so that the activity could begin only after prolonged periods of hesitation.

The third part of the LTP, instead, is the one that specifically requires the interaction to assume a triadic configuration (3-together). In the IA-Group it was possible to notice that the transition to this part often occurred when the difficulties in the configuration (2 + 1) became intensely pronounced, as if the intervention of a third active part could provide more sustain to the ongoing interaction. Unfortunately, it was rare for the subjects to benefit from this kind of “support.” Family 4, for example was able to move initially to a triadic configuration but subsequently the interaction got confused and lasted too long, going to the detriment of the fourth part. In family 2, instead, the abrupt transition enhanced the previous difficulties and required to move almost immediately to the last phase, not allowing the family to establish a clear and shared triadic joint activity. These examples suggest that interacting in a triadic way might be particularly challenging for the IA-Group. At the same time, they highlight the presence of differences that characterize each family, suggesting the importance to consider the specific sequence of each LTP procedure and how it is structured, since the choices made by the family are inscribed into specific family relational structures.

Thirdly, when referring to more defined aspects of triadic interactions, the IA-Group seemed to show specific difficulties in the bodily creation of a triangular space able to allow familial interactions (through the adoption of adequate gazes and body postures) and in the sharing and validation of emotional states (both of the child and of the family system).

The partners repeatedly alternated bodily signals that showed “readiness to interact” and a focus on the task, and moments where they clearly displayed signs of unavailability and disengagement; the body could be oriented elsewhere and the interaction could lack of eye contact. Depending on the family, these misalignments could be shown more intensely by the parents, for example by focusing their attention out of the triangular space, or by the children; some children did not orient their gaze, the torso or the hips toward their interactive partner for prolonged periods, whereas other children, instead, physically moved away from the interaction area during the most stressful times.

Rarely the emotional climate of the LTP appeared clearly positive or pleasant; at times the atmosphere seemed tense. In family 4 and 5, for example, it was possible to observe that affects could be shared only by dyadic sub-systems, while the third partner often appeared emotionally incoherent (for example with the mother being very serious while the father and the child were enjoying an activity together, or vice versa).

In addition, carrying out adequate repairs and resolutions of the possible interactive mistakes emerging during shared activities appeared to be another challenge for these families. Moreover, when referring specifically to the Feeding context, difficulties inherent the co-construction of a joint activity seemed to emerge, pointing out difficulties of the triads to coordinate and cooperate with each other. It appeared hard, for the partners, to build playful exchanges on a joint activity. On different occasions, the parents struggled and failed to coordinate together in order to carry on the play or the meal. At times, this seemed to depend on the contrast and the incoherence between the different individual approaches that the parents adopted when interacting with the child (for example with one partner being very stimulating, at times rather intrusive, and the other one being very passive, and under-stimulating). In other cases, this was due to a lack of mutual ratifications of the parents’ suggestions. On other occasions, the difficulties in co-constructing and in repairing interactive mismatches appeared enhanced by the child’s responses toward parental non-optimal interactive bids. For example, in family 3 and 4 the parents showed clear difficulties in following their children’s initiatives and in respecting adequate timing during the activities, resulting in an increase in the child’s distress. When intensely distressed, the children markedly refused the adults’ initiatives, with intense protests and complaints that made it hard to reach an adequate repair of the interactive ruptures.

This last aspect could be linked to our finding that highlights how children with IA showed poor social competence for age, little autonomy and difficulties in self-regulation, and in the possibility to be engaged in the interaction with both parents in a developmentally adequate way. These results confirm previous research (Stein et al., 1999; Cooper et al., 2004; Ammaniti et al., 2010, 2012; Lucarelli et al., 2012, 2013) on the dysfunctional developmental trajectories in children diagnosed with IA, further showing the possible impact of the lower quality of triadic parents–child interactions in the emotional and social development of these children. More in particular, despite their age, in fact, some children preferred to be fed by their parents, rather than eating alone, as it happened for the young boy in family 4. Moreover, the increasing tensions in family interactions, and the difficulties of the parents in managing negative emotions, appeared to compromise the children’s self-regulation competencies, making it hard to modulate their internal state. Despite their parents’ attempts, emotion regulation was often not successful on these occasions, with the result that the incremental distress jeopardized the organization of affects and behaviors for the children, compromising also their possibility to maintain an adequate involvement in familial activities. This difficulty could be expressed differently from the children; some adopted more subtle ways to disengage from the interaction, as it happened for the little girl in family 2, who remained “physically” present in the triangular space but focusing her attention elsewhere, excluding the adults, whereas other children were more likely to actively refuse to carry on the activities.

Taken together, the results of our pilot study pointed out the lower quality of triadic parents–child interactions in families of children diagnosed with IA, when compared to matched families with normally developing children. These families seem more likely to experience difficulties in affective attunement, and in the sharing of pleasure and of emotional co-constructed states during interactions, specifically in the context of feeding interactions. These difficulties appear to involve specific domains of family functioning, as evidenced by the lower scores on the FAAS variables. Anyway, as highlighted above, each family expresses them in its unique way. Overall, these observations seem in line with the extant literature and suggest that dysfunctional family interactions are a critical issue for IA that affects dyadic, co-parental and family subsystems, stressing the importance of an articulated diagnostic assessment in order to target effective treatment approaches.

Although it represents a novelty in the clinical field explored, being a pilot study, our current research shows also a series of limitations which could offer useful suggestions to implement future research. The first limit regards the sample; the small amount of participants prevent us to generalize the obtained results. Anyway, these data appear in line with previous studies highlighting the presence of interactive difficulties specifically ascribable to the feeding context in families with children diagnosed with IA. In addition to the theoretical and empirical literature that highlights the strong associations between maternal features and IA (Stein et al., 1999; Chatoor et al., 2000; Ammaniti et al., 2004b, 2010; Lucarelli et al., 2013), our results seem to suggest that it is not only the mother–child but also the mother–father–child interactions, and the triadic configuration mother–father–child taken together as a system, which are at risk in these families. Therefore, although fathers do not usually bear the primary feeding responsibility, they should be given more consideration in the diagnostic and intervention process of infantile feeding disorders. Raising professional awareness to the role of fathers as well as to the understanding that through their feeding disorder children reflect their relationship difficulties with both parents may lead to the construction of specific interventions that focus not only on the mother–child relationship but also on the co-parental and family subsystems to strengthening early relationships in at-risk populations. Moreover, an attempt to overcome this limit was carried out through the adoption of a control group, that allowed us to make some preliminary hypotheses about the specificity of the difficulties detected during triadic interactions in families with children diagnosed with IA through the comparison with families that did not show the presence of feeding disturbances and where the children were normally developing.

A second limit concerns an issue which is more of methodological concern, i.e., the fact that this was the first study to specifically apply the LTP procedure to the feeding context. In this sense, it would be desirable, in the future, to replicate this research design in wider samples, in order to be able to test with more statistical strength the psychometric properties of the LTP procedure adapted to the feeding context. Anyway, as far as it concerns out study, the LTP showed to be a procedure flexible enough to be applied to a context different from the one of Play (i.e., the Feeding context) and the adaptations of setting and assignments (i.e., the request to interact in a triadic way focusing on a meal rather than on toys or games) appeared feasible and well-tolerated by the families This finding appears in line with other studies (Hedenbro et al., 2006; Miscioscia et al., 2013; Gatta et al., 2014, 2015) that already suggested that adaptations of the LTP do not seem to compromise the internal consistency and the reliability of the coding system and of the procedure itself. In this sense, the preliminary results of our pilot study seem promising in further encouraging the adoption of this procedure in order to reach a better understanding of the links between triadic family functioning and feeding disorders in infancy.

## Author Contributions

LL, MA, and AS prepared the study design, and supervised the research team; LL wrote the introduction and the discussion sections of the manuscript and recruited the clinical sample; MA supervised the preparation of the manuscript, contributing to the writing of the introduction and the discussion sections; AS recruited the normative sample and supervised the preparation of the manuscript, contributing to the writing of the methods and the discussion sections; AP prepared data set, performed statistical analyses, prepared tables and figures, and contributed to write the discussion section. All authors reviewed the manuscript.

## Conflict of Interest Statement

The authors declare that the research was conducted in the absence of any commercial or financial relationships that could be construed as a potential conflict of interest.

The reviewer GDF declared a shared affiliation, though no other collaboration, with the author MA to the handling Editor, who ensured that the process nevertheless met the standards of a fair and objective review.

## Footnotes

1 See Favez et al. (2011) for a more detailed description of the scales, or Lucarelli et al. (2012) and Simonelli et al. (2016) for the application of the FAAS to the Italian population.

## References

[B1] Ammaniti M. Ambruzzi A. M. Lucarelli L. Cimino S. D’Olimpio F. (2004a). Malnutrition and dysfunctional mother-child feeding interactions: clinical assessment and research implications. *J. Am. Coll. Nutr.* 23 259–271. 10.1080/07315724.2004.10719369 15190051

[B2] Ammaniti M. Lucarelli L. Cimino S. D’Olimpio F. (2004b). Transmission intergenerationnelle: troubles alimentaires de l’enfance et psychopathologie maternelle. *Devenir* 16 173–198. 10.3917/dev.043.0173

[B3] Ammaniti M. Gallese V. (2014). *The Birth of Intersubjectivity: Psychodynamics, Neurobiology, and the Self.* New York, NY: W.W. Norton & Company.

[B4] Ammaniti M. Lucarelli L. Cimino S. D’Olimpio F. Chatoor I. (2010). Maternal psychopathology and child risk factors in infantile anorexia. *Int. J. Eat. Disord.* 43 233–240. 10.1002/eat.20688 19350650

[B5] Ammaniti M. Lucarelli L. Cimino S. D’Olimpio F. Chatoor I. (2012). Feeding disorders of infancy: a longitudinal study to middle childhood. *Int. J. Eat. Disord.* 45 272–280. 10.1002/eat.20925 21495054

[B6] Atzaba-Poria N. Meiri G. Millikovsky M. Barkai A. Dunaevsky-Idan M. Yerushalmi B. (2010). Father-child and mother-child interactions in families with a child feeding disorder: the role of paternal involvement. *Infant Ment. Health J.* 31 682–698. 10.1002/imhj.20278 28543067

[B7] Barriguete M. J. A. Lebovici S. Salinas J. L. Moro M. R. Solis L. Botbol M. (2002). “Les fonctiones du père. Troubles de la conduite alimentaire dans la clinique des interactions précoces,” in *La Parentalité* ed. Solis L (Paris: PUF) 73–90.

[B8] Benoit D. (2000). “Feeding disorders, failure to thrive, and obesity,” in *Handbook of Infant Mental Health* ed. Zeanah C. H. (New York, NY: The Guilford Press) 339–352.

[B9] Bryant-Waugh R. Markham L. Kreipe R. E. Walsh B. T. (2010). Feeding and eating disorders in childhood. *Int. J. Eat. Disord.* 43 98–111. 10.1002/eat.20795 20063374

[B10] Carneiro C. Corboz-Warnery A. Fivaz-Depeursinge E. (2006). Prenatal coparenting and postnatal family alliance. *Infant Ment. Health J.* 27 207–228. 10.1002/imhj.20089 28640414

[B11] Chatoor I. (2009). *Diagnosis and Treatment of Feeding Disorders in Infants, Toddlers, and Young Children.* Washington, DC: Zero To Three/National Center for Clinical Infant Programs.

[B12] Chatoor I. Ganiban J. Hirsh R. Borman-Spurrell E. Mrazek D. A. (2000). Maternal characteristics and toddler temperament in infantile anorexia. *J. Am. Acad. Child Adolesc. Psychiatry* 39 959–967. 10.1097/00004583-200312000-00018 10846309

[B13] Chatoor I. Ganiban J. Surles J. Doussard-Roosevelt J. (2004). Physiological regulation and infantile anorexia: a pilot study. *J. Am. Acad. Child Adolesc. Psychiatry* 43 1019–1025. 10.1097/01.chi.0000126977.64579.4e 15266197

[B14] Cimino S. Cerniglia L. Porreca A. Simonelli A. Ronconi L. Ballarotto G. (2016). Mothers and fathers with binge eating disorder and their 18-36 months old children: a longitudinal study on parent-infant interactions and offspring’s emotional-behavioral profiles. *Front. Psychol.* 7:580 10.3389/fpsyg.2016.00580 PMC4843107 27199815

[B15] Cooper P. J. Whelan E. Woolgar M. Morrell J. Murray L. (2004). Association between childhood feeding problems and maternal eating disorders: role of the family environment. *British J. Psychiatry* 184 210–215. 10.1192/bjp.184.3.210 14990518

[B16] Fadda R. Lucarelli L. Parisi M. (2014). Interazioni madre-bambino e competenze socio-comunicative nell’infanzia. *Psicol. Clin. Dello Sviluppo* 18 377–401. 10.1449/78364

[B17] Favez N. Lopes F. Bernard M. Frascarolo F. Scaiola C. L. Corboz-Warnery A. (2012). The development of family alliance from pregnancy to toddlerhood and child outcomes at 5 years. *Fam. Process* 51 542–556. 10.1111/j.1545-5300.2012.01419.x 23230984

[B18] Favez N. Scaiola C. L. Tissot H. Darwiche J. Frascarolo F. (2011). The family alliance assessment scales: steps towards validity and reliability of an observational assessment tool for early family interactions. *J. Child Family Stud.* 20 23–37. 10.1007/s10826-010-9374-7

[B19] Feldman R. (2007). Parent-infant syncrony. Biological foundations and developmental outcomes. *Curr. Dir. Psychol. Sci.* 16 340–345.

[B20] Fivaz-Depeursinge E. (2008). ‘Infant’s triangular communication in ‘two for one’ versus ‘two against one’ family triangles: Case illustrations. *Infant Ment. Health J.* 29 189–202. 10.1002/imhj.20174 28636106

[B21] Fivaz-Depeursinge E. Corboz-Warnery A. (1999). *The Primary Triangle: A Developmental System View of Mothers, Fathers, and Infants.* New York, NY: Basic Books.

[B22] Fivaz-Depeursinge E. Favez N. Lavanchy S. De Noni S. Frascarolo F. (2005). Four-month-olds make triangular bids to father and mother during trilogue play with still-face. *Soc. Dev.* 14 361–378. 10.1111/j.1467-9507.2005.00306.x

[B23] Fivaz-Depeursinge E. Lavanchy-SCaiola C. Favez N. (2010). The young infant’s triangular communication in the family: access to threesome intersubjectivity? Conceptual considerations and case illustrations. *Psychoanal. Dialogues* 20 125–140. 10.1080/10481881003716214

[B24] Fonagy P. Gergely G. Jurist E. L. Target M. (2002/2005). *Regolazione Affettiva, Mentalizzazione e Sviluppo del Sé.* Milano: Tr. it. Raffaello Cortina Editore.

[B25] Gatta M. Simonelli A. Sudati L. Sisti M. Svanellini L. Stucchi M. (2015). Emotional difficulties in adolescence: psychopathology and family interactions. *Int. Neuropsychiatr. Dis. J.* 4 47–54. 10.9734/INDJ/2015/17789

[B26] Gatta M. Sisti M. Brunello G. Sale E. Simonelli A. Battistella P. A. (2014). Valutazione delle relazioni familiari nell’intervento psicomotorio con pre-adolescenti affetti da paralisi cerebrale. *G. Neuropsichiatr. dell’Età Evol.* 34 73–81.

[B27] Goodlin-Jones B. L. Anders T. F. (2001). Relationship disturbances and parent-child therapy. Sleep problems. *Child Adolesc. Psychiatr. Clin. N. Am.* 10 487–499. 11449808

[B28] Hall K. M. R. Simonelli A. Viola A. (2014). “Feeding development, father involvement and family interactions: comparison of two single cases,” in *Proceedings in Scientific Conference SCIECONF* Žilina 223–228.

[B29] Hedenbro M. Shapiro A. F. Gottman J. M. (2006). Play with me at my speed: describing differences in the tempo of parent-infant interactions in the Lausanne Triadic Play paradigm in two cultures. *Fam. Process* 45 485–498. 10.1111/j.1545-5300.2006.00184.x 17220116

[B30] Kuczmarski R. J. Ogden C. L. Grummer-Strawn L. M. Fegal K. M. Guo S. S. Mei Z. (2000). CDC growth charts: United States. *Adv. Data* 314 1–27. 11183293

[B31] Lieberman A. F. Slade A. (2000). “Parenting toddlers: developmental and clinical considerations,” in *WAIMH Handbook of Infant Mental Health: Parenting and Child Care* Vol. 3 eds Osofsky J. D. Fitzgerald H. E. (New York, NY: J Wiley & Sons) 25–56.

[B32] Lucarelli L. Cimino S. D’Olimpio F. Ammaniti M. (2013). Feeding disorders of early childhood: an empirical study of diagnostic subtypes. *Int. J. Eat. Disord.* 46 147–155. 10.1002/eat.22057 23015314

[B33] Lucarelli L. Speranza A. M. (2014). “The relationship between maternal attachment/psychopathology and the severity of Infantile Anorexia,” in *Proceedings of the 14th Congress of World Association for Infant Mental Health: Infant Mental Health Journal* Vol. 35 Edinburgh 106–107.

[B34] Lucarelli L. Simonelli A. Ammaniti M. (2012) “Infantile anorexia: dyadic and triadic interactions during feeding and play,” in *Proceedings of the 13th Biennial World Congress Cape Town; Infant Mental Health Journal* Vol. 33 Cape Town 6.

[B35] Lyons-Ruth K. Zeanah C. H. Benoit D. (1996). “Disorders and risk for disorder during infancy and toddlerhood,” in *Child Psychopathology* eds Mash J. Barkley R. A. (New York, NY: The Guilford Press) 457–491.

[B36] McHale J. Fivaz-Depeursinge E. Dickstein S. Robertson J. Daley M. (2008). New evidence for the social embeddedness of infants’ early triangular capacities. *Fam. Process* 47 445–463. 10.1111/j.1545-5300.2008.00265.x 19130787 PMC2761722

[B37] McHale J. P. (1995). Coparenting and triadic interactions during infancy: the roles of marital distress and child gender. *Dev. Psychol.* 31 985–996. 10.1037/0012-1649.31.6.985

[B38] McHale J. P. (2007). When infants grow up in multiperson relationship systems. *Infant Ment. Health J.* 28 370–392. 10.1002/imhj.20142 21512615 PMC3079566

[B39] Minuchin S. (1974). *Families and Family Therapy.* Cambridge, MA: Harvard University Press.

[B40] Miscioscia M. D’Amore S. Delvoye M. (2013). From two to three: transition to parenthood and family alliance in families with lesbian parents. *Thér. Fam.* 34 131–148. 10.3917/tf.131.0131

[B41] Murray L. (2014). *The Psychology of Babies. How Relationships Support Development from Birth to Two.* London: Constable & Robinson Ltd. 10.1080/02646838.2016.1186267 29517287

[B42] Nadel J. Tremblay-Leveau H. (1999). “Early perception of social contingencies and interpersonal intentionality: dyadic and triadic paradigms,” in *Early Social Cognition: Understanding others in the First Month of Life* ed. Rochat P. (Mahwah, NJ: Lawrence Erlbaum Associates Publishers) 189–212.

[B43] Sameroff A. (2010). A unified theory of development: a dialectic integration of nature and nurture. *Child Dev.* 81 6–22. 10.1111/j.1467-8624.2009.01378.x 20331651

[B44] Simonelli A. Bighin M. De Palo F. (2012). Coparenting interactions observed by the prenatal Lausanne Trilogue Play: an Italian replication study. *Infant Ment. Health J.* 33 609–619. 10.1002/imhj.21350 28520113

[B45] Simonelli A. De Palo F. Bighin M. (2014). From pregnancy to the 9th month: the development of early interactive-relational competencies of the child in the family context. *Interdiscip. J. Fam. Stud.* 19 110–130. 10.13140/2.1.4110.1442

[B46] Simonelli A. Fava Vizziello G. Bighin M. Petech E. (2010). Co-regulation processes and development of triadic competences in the first year of infant’s life. *Psicol. Clin. dello Sviluppo* 14 527–544. 10.1449/33628

[B47] Simonelli A. Parolin M. Sacchi C. De Palo F. Vieno A. (2016). The role of father involvement and marital satisfaction in the development of family interactive abilities: a multilevel approach. *Front. Psychol.* 7:1725 10.3389/fpsyg.2016.01725 PMC5098289 27872601

[B48] Stein A. Woolley H. McPherson K. (1999). Conflict between mothers with eating disorders and their infants during mealtimes. *Br. J. Psychiatry* 175 455–461. 10.1192/bjp.175.5.455 10789278

[B49] Stern D. (1996). “Babies and music: some reflections on the temporal aspects of an infant daily experience,” in *Le temps et la forme. Cahier de la Faculté des Lettres, Musicologie* ed. Darbellay E. (Genève: Université de Genève) 167–189.

[B50] Stern D. N. (1985). *The Interpersonal World of the Infant. A View from Psychoanalysis and Developmental Psychology.* New York, NY: Basic Books.

[B51] Stern D. N. (2010). *Forms of Vitality: Exploring Dynamic Experience in Psychology, the Arts, Psychotherapy, and Development.* Oxford: Oxford University Press.

[B52] Tremblay-Leveau H. (1999). Avant les croyances. *Enfance* 52 313–321.

[B53] Tremblay-Leveau H. Nadel J. (1995). Young children’s communication skills in triads. *Int. J. Behav. Dev.* 18 227–242.

[B54] Waterlow J. C. Buzina R. Keller W. Lan J. M. Nichaman M. Z. Tanner J. M. (1977). The presentation and use of height and weight data for comparing the nutritional status of groups of children under the age of 10 years. *Bull. World Health Organ.* 55 489–498. 304391 PMC2366685

[B55] Zero To Three (2005). *Diagnostic Classification of Mental Health and Developmental Disorders of Infancy and Early Childhood (Revised).* Arlington, VA: Zero To Three/National Center for Clinical Infant Programs.

